# Preclinical models of orthopaedic trauma: Orthopaedic Research Society (ORS) and Orthopaedic Trauma Association (OTA) symposium 2022

**DOI:** 10.1097/OI9.0000000000000303

**Published:** 2024-03-14

**Authors:** Patrick M. Wise, Augustine M. Saiz, Justin Haller, Joseph C. Wenke, Thomas Schaer, Prism Schneider, Saam Morshed, Chelsea S. Bahney

**Affiliations:** a Department of Orthopaedic Surgery, University of California, Davis, Sacramento, CA; b Department of Orthopaedic Surgery, University of Utah, Salt Lake City, UT; c Department of Orthopaedic Surgery and Rehabilitation, University of Texas Medical Branch at Galveston, Galveston, TX; d Shriners Children's Texas, Galveston, TX; e Department of Clinical Studies, New Bolton Center University of Pennsylvania School of Veterinary Medicine, Kennett Square, PA; f Section of Orthopaedic Surgery, Department of Surgery, University of Calgary, Calgary, AB, Canada; g Orthopaedic Trauma Institute, University of California, San Francisco (UCSF), San Francisco, CA; h Center for Regenerative and Personalized Medicine, The Steadman Clinic & Steadman Philippon Research Institute, Vail, CO

**Keywords:** trauma, fracture, infection, translational research, animal models, basic science, preclinical

## Abstract

Orthopaedic trauma remains a leading cause of patient morbidity, mortality, and global health care burden. Although significant advances have been made in the diagnosis, treatment, and rehabilitation of these injuries, complications such as malunion, nonunion, infection, disuse muscle atrophy and osteopenia, and incomplete return to baseline function still occur. The significant inherent clinical variability in fracture care such as differing patient demographics, injury patterns, and treatment protocols make standardized and replicable study, especially of cellular and molecular based mechanisms, nearly impossible. Hence, the scientists dedicated to improving therapy and treatments for patients with orthopaedic trauma rely on preclinical models. Preclinical models have proven to be invaluable in understanding the timing between implant insertion and bacterial inoculation on the bioburden of infection. Posttraumatic arthritis (PTOA) can take years to develop clinically, but with a porcine pilon fracture model, posttraumatic arthritis can be reliably induced, so different surgical and therapeutic strategies can be tested in prevention. Conversely, the racehorse presents a well-accepted model of naturally occurring PTOA. With preclinical polytrauma models focusing on chest injury, abdominal injury, multiple fractures, and/or head injury, one can study how various injury patterns affect fracture healing can be systemically studied. Finally, these preclinical models serve as a translational bridge to for clinical application in human patients. With selection of the right preclinical model, studies can build a platform to decrease the risk of emerging technologies and provide foundational support for therapeutic clinical trials. In summary, orthopaedic trauma preclinical models allow scientists to simplify a complex clinical challenge, to understand the basic pathways starting with lower vertebrate models. Then, R&D efforts progress to higher vertebrate models to build in more complexity for translation of findings to the clinical practice.

## 1. Introduction

The Basic Science Focus Forum of the 2022 Orthopaedic Trauma Association meeting featured the “OTA/AO and ORS International Section for Fracture Repair (ISFR) Collaborative Basic Science Forum Workshop: Preclinical Models of Orthopaedic Trauma” to discuss animal models and their translation to patients with orthopaedic trauma. The goal of the focus forum was to provide advice and outline a plan for orthopaedic surgeons interested in developing translational research and provide examples of previous animal models that have been successful. Specifically, these experts discussed animal models involving fracture-related infections, posttraumatic osteoarthritis, and nonunion/delayed fracture healing after multiple trauma.

## 2. From Mice to Human: Choosing the Right Animal Model to Facilitate Clinical Translation

**Figure 1.**
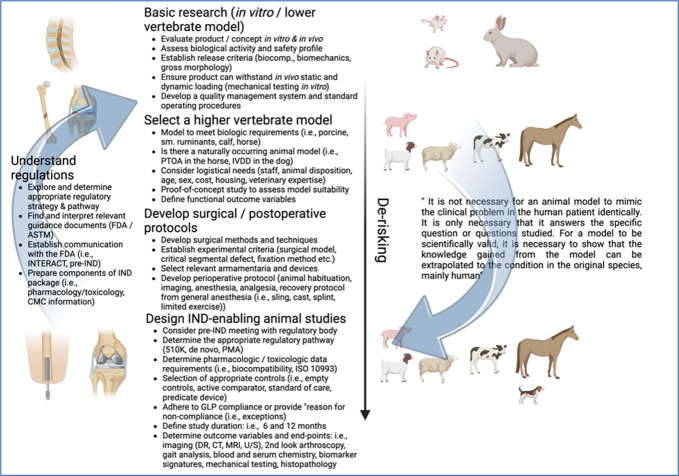
Key milestones in translational research/commercialization from basic research to clinical trials. Researchers must navigate the many challenges when transitioning from in vitro research to lower vertebrate animal model selection (rodent, rabbit), followed by higher vertebrate models (small ruminant, pig, calf, horse) and protocol development, including IND-enabling animal studies. All stages should be executed consistent with appropriate regulations and guidance (FDA/ASTM guidance documents, IACUC, ARRIVE guidelines, GLP). 510K/PMA: 510(k) clearance is authorization from the FDA to market a medium-risk medical device, while PMA (premarket approval) may be required for more high-risk and novel products. Created with BioRender.com. ARRIVE, Animal Research: Reporting of In Vivo Experiments; CMC, chemistry, manufacturing, and controls; FDA, Food and Drug Administration; GLP, good laboratory practice; IACUC, Institutional Animal Care & Use Committee; IND/IDE, investigational new drug/investigational new device; INTERACT, initial targeted engagement for regulatory advice on CBER products.

Research and product/treatment design can be separated into 3 main phases (Fig. 1).

1. *Discovery research*: Studies that explore hypothesis-driven mechanistic questions. They can lead to developing intellectual property and patent filing.
2. *Translational research leading toward commercialization*: There is typically a value proposition at the core that addresses an unmet clinical need. It starts with proof-of-concept studies to attract funding. The derisking process continues as the technology readiness level increases with the goal of regulatory approval.
3. *Product launch:* Market approval, reimbursement is as an important milestone in product development as is regulatory approval.

The primary question that deserves to be addressed is why we need to use animal models for investigating orthopaedic problems and their potential solutions. One may argue that with all the technology and advances made using cell and tissue cultures, robotic biomechanical testing, and computer modeling, animal testing may no longer be necessary. Although these types of studies are very useful for providing critically important preliminary data for screening, prioritizing and optimizing mechanisms, drugs, devices, and biologics for potential clinical application, they are not sufficient for determining safety and efficacy for use in human or veterinary patients. For these reasons, regulatory bodies such as the Food and Drug Administration require well-designed, ethical animal model studies that provide valid preclinical data before any clinical implementation of these methods and products. Animal models provide clinically applicable data that enhance the likelihood for safe and effective outcomes in clinical trials.

In preparation for the transition from in vitro to in vivo animal models, selection of the correct animal model is imperative. There are several factors that go into this decision, and investigators can choose between lower vertebrate models and higher vertebrate models. Rats, mice, or rabbits are the most commonly used vertebrate models, as they are less expensive and easier to obtain/care for in large numbers, whereas the larger animal models such as horses, sheep, pigs, or dogs provide more realistic models to humans (Fig. 1). It is obvious that any associated model limitations provide the scientific context when discussing results or prospective clinical utility in the target patient (human).There is however a commercialization “lull” that can occur at the early stages of translational research between government funding and venture capital or industry support for prototypes and products.^1^ In order to obtain funding and capital investment, risk reduction is key by showing the product/treatment can obtain intellectual property protection or can be trademarked, it is safe and works, can be manufactured competitively, has the potential to create clinical value. To do so, methodological in vitro and in vivo testing is required to demonstrate biological evidence that this material works and that the transition from in vitro to in vivo is possible.^2^

Finally, the transition to more clinically relevant models (size, geometry, mechanical loading etc.) may be appropriate starting with proof-of-concept studies that validate safety, support efficacy for intended indications, and provide data for determining appropriate sample size for pivotal studies designed to address scientific, regulatory, and market-based milestones toward market launch or clinical trials. This can progress to a pivotal study, and depending on the nature of the study include the use of skeletally mature large animals with a defect or lesion that has been validated such that it is symptomatic and will not spontaneously resolve. Pivotal studies need to have robust and relevant controls and/or cohort groups, be of at least 6-month duration and include diagnostic imaging, clinical, functional, gross, biomechanical, and histologic outcome measures and be performed consistent with good laboratory practice (CFR21part 58) regulations (Fig. 1).

Additional consideration is required for understanding how the animals will react to the disease and/or treatment. Preclinical animal models demonstrate differences in adaptative weight-bearing after limb surgery which becomes critically important during the postoperative phase in clinical applicability.^3^ For instance, to study hardware augmentation as it relates to fragility fractures and early mobilization in the elderly, that is, pertrochanteric femur fracture, the choice of the dog model would demonstrate less translational fidelity as dogs normally just adapt to walk on 3 legs when 1 is painful. Pigs, on the other hand, tend to want to ambulate on 4 legs and would consequently be forced to weight bear immediately on the operated leg with the augmented hardware and therefore provide a test system of greater clinical utility.

Throughout this process, using test systems of lower or higher vertebrate animals, researchers must follow the relevant animal welfare guidelines of their respective institutions and importantly the recently established ARRIVE guidelines (Animal Research: Reporting of In Vivo Experiments) now often required for publications in scientific journals (https://arriveguidelines.org/). Large animal translational research requires an expert, passionate, and well-coordinated team.^4,5^ The animal–human bond is one that will inevitably form between caregivers and test subjects. This bond gets constantly disrupted due to the nature of terminal studies in experimental models. Compassion fatigue is the number one reason for animal care staff to quit their assignments, and it is important to be mindful of this and provide a transparent support system where mourning is accepted, even encouraged. A simple memorial wall from animals of past studies can provide a platform to honor and value the contribution of research animals to biomedical research and the advancements in medical care.

## 3. Animal Models for Nonunion and Delayed Fracture Healing After Multiple Trauma

There is an obvious difference in healing between a patient who sustained an isolated ankle fracture compared with a patient with chest/brain trauma and multiple fractures requiring damage control orthopaedics and possibly multiple procedures. This is due to a variety of processes within the human body including a posttrauma inflammatory cascade, a host defense response that affects fracture healing, plasmatic cascade system, capillary leakage due to oxidative stress and anaerobic metabolism, and ischemia/reperfusion injury.^6^ Unfortunately, investigating this in a human model can be difficult, while also caring for them clinically. Furthermore, the polytrauma patient population is heterogenous, with patients sustaining a variety of different injuries, having different surgical interventions at different times, and variable demographics and preinjury medical conditions. Preclinical polytrauma models allow us to isolate certain processes and study them extensively.

**Figure 2.**
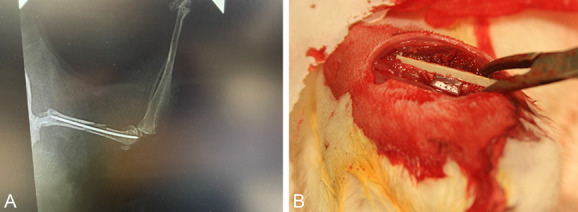
A, Mouse femur fracture with intramedullary pin for study of fracture healing in the setting of polytrauma. B, A rat femur segmental defect treated with plate and biomaterial (alginate hydrogel with mesenchymal stem/stromal cells) to assess fracture healing in setting of muscle injury.

There are numerous polytrauma animal models that have been studied to date (Fig. 2). Recknagel et al^7^ created a rodent model that examined fracture healing in the setting of concomitant chest trauma. This study demonstrated that increased systemic and local inflammation after multiple trauma (ie, chest injury combined with fracture) is associated with nonunion/delayed union. Similarly, in a rodent model of chest trauma, dorsal burn, and fractures, Mangum et al^8^ again showed delayed union in the polytrauma group, in addition to a difference in white blood cell (WBC) levels, concentration of inflammatory cytokines, and different expression of genes compared with the control group.

Marioka et al^9^ and Mollahosseneini et al^10^ examined rodents that sustained head injuries and fractures and found systemic cytokine changes important to fracture healing. Stress alone has been shown to affect fracture healing in the rodent model studied by Haffner-Luntzer et al.^11^ In this model, younger mice were stressed by an alpha mouse, creating a catecholamine response within them that inhibited fracture healing. In larger vertebrate porcine model, changes in microcirculation, systemic and local cytokine composition, and gene/protein/microRNA expression were shown to affect fracture healing after chest trauma, liver injury, and hemorrhagic shock.^12–14^

Regarding translation, currently therapeutic pathways are being investigated by groups to develop pharmacological targets that promote fracture healing in polytrauma patients.^15,16^ Other groups are examining cytokines as markers of injury severity in patients to test the hypothesis that composition, levels, and timing of cytokine profiles will serve as predictors of trauma severity, healing potential, and dictate when to operate.^17–20^ The in vivo work to date is promising, and while there is still much more to be done, translatable animal models will serve as an important step.

## 4. Fracture-Related Infection: Preclinical Models to Assess Emerging Therapies

When creating or using an animal model, the goal should not be to fully mimic the patient or condition. Rather, the model must possess the critical aspect or challenge that causes the standard of care to fail in patients. Whether studying prevention of surgical site infections, treatment for acute contaminated fractures, chronic osteomyelitis, or implant-related infections, each condition has a reason why infection occurs or is recalcitrant to current therapies; a deep understanding of the problem and limitations of the current standard of care is needed to select both the best preclinical model and experimental design.

Determining which species, strain, inoculum amount, and duration from contamination to treatment are all important considerations given their influence on outcomes. For example, in an infection fracture rat model, a segmental rodent femur defect was created, stabilized, and then infected with *Staphylococcus aureus*. When the defect was infected with just a 1 × 10^2^ colony forming units concentration, only 50% of the bones/implants became infected compared with 100% when 1 × 10^3^ colony forming units or more concentration was used.^21^ Counter to this, in certain situations, the inoculation amount may be too high and not model the specific human condition appropriately. For example, when looking at implant-related infections, if you inoculate the animal with too many bacteria, the typical battle between body and bacteria will not take place and biofilm will just form immediately and the treatment that is being evaluated will fail.

Furthermore, the time from inoculation to treatment affects the outcome of treatments and can be used to create the appropriate criteria for evaluating therapies that have different mechanisms of action. For instance, vancomycin is effective against planktonic bacteria but not biofilm. Treatment with topical vancomycin powder at 6 hours, which is before the bacteria become tolerant, is effective at reducing bacterial load, but treatment after 24 hours does not eradicate the bacteria.^22^ However, treatment with rifampin, which is also effective against *S. aureus* in a biofilm, works both at 6 and 24 hours postcontamination.^23^ Modifying different aspects to create the appropriate and relevant challenge is critical.

There is generally a suitable preclinical model that can be used or slightly modified to address your question, but when there is not, the investigator must create one. Surface coating and modifications have been a very active focus for decades as the goal is to prevent bacteria from being able to attach and form biofilms on these materials. This race between bacteria colonizing implants before the host can integrate can lead to infection. Unfortunately, a time frame for how long it takes for the host cells to be able to thwart infection was not known making it more unnecessarily challenging to design implant coatings. An implant-related infection model for this exact purpose was developed by uncoupling the placement of the implant (metal K-wire in femoral canal) and bacterial challenge; *S. aureus* was administered intravenously at various times after placement of implant. From this, it was determined that it takes approximately 7 days for the host to prevent infection from occurring; as expected, it took this long for a meaningful number of immune cells to be present on the implant.^24^ This model can now be used to evaluate various implant coating approaches.

Understanding the clinical problem and why the standard of care fails along with clearly defining the question being asked will serve as a roadmap and guide for selection or creation of the ideal preclinical model.

## 5. Posttraumatic OA: Current Models and Translational Relevance

For all large and small animal models, including models for posttraumatic osteoarthritis, there are a variety of requirements, as described by Little et al. First, you must induce a reproducible disease that occurs in a suitable time frame; you must encompass a progressive time frame to allow early, mid, and late investigation; and you must select a mammalian animal that allows multiple analyses and genome/proteomic analysis. Furthermore, the disease process must be similar to human pathology and involve all tissues, and ultimately, the animal model should allow for similar therapeutic disease modification as in humans.^25^

There are many pros and cons for small animal posttraumatic arthritis models. On the positive side, they are relatively cheap, animal housing is streamlined, anesthesia is easy, and whole joint sample acquisition is possible. Furthermore, there is little species variation, they are genetically modifiable, and there are readily available assays. Unfortunately, downsides of the small animal model include difficulty obtaining synovial fluid/biomarkers, fixing/instrumenting pathology, and injecting therapeutics due to size. Small animals also lack true skeletal maturity, so there is always some level of physeal activity, they also possess a different cartilage composition than humans, and typically your joints selection is limited as you can only really use the knee.

Several small animal models currently exist for posttraumatic osteoarthritis. Glasson et al^26^ compared anterior cruciate ligament (ACL) transection ± destabilization of the medial meniscus with just destabilization of the medial meniscus alone. The ACL transection model resulted in significant osteoarthritis, but it required a higher surgical proficiency, was rapid severe osteoarthritis, was invasive, and represented a persistent instability model. In comparison, the destabilization of the medial meniscus model was minimally invasive, led to a gradual progression of osteoarthritis over time, and had consistency with normal age-related arthritis. Ultimately, the destabilization of the medial meniscus model was deemed as the preferred model compared with ACL transection model. However, even if the disease phenotype, here posttraumatic osteoarthritis, is reliably produced, the “trauma” may not be similar to the articular fracture mechanism that might be of interest.

Several noninvasive models also exist that range from intra-articular fracture of tibial subchondral bone, cyclic tibial compression loading of articular cartilage, and ACL rupture through tibial compression overload.^27^ Rai et al subjected the right knee of mice to axial tibial compression with 3 separate loading magnitudes were applied (6N, 9N, and 12N). This allowed for progressive PTOA development overtime, increased severity of PTOA with increased force, and produced a reproducible cartilage/ligament injury; however, this model is not as translatable to humans since the posttraumatic condition was not fixed.^28^

An example of a more translatable model was performed by Wei et al.^29^ In their model, rabbits underwent closed joint trauma to produce ACL ruptures and meniscus damage, and then, unlike previously mentioned models, the traumatized knee was surgically repaired using a semitendinosus ACL autograft. Despite repair, the knees damaged and subsequently repaired demonstrated a progressive degeneration overtime. This model is easily translatable and may play a role in the development of future PTOA interventions.

There are also a variety of advantages and disadvantages for large animal posttraumatic arthritis models. Compared with the smaller counterparts, larger animals have more similar cartilage composition to humans, their pathology is easier to treat, it is easier to obtain synovial fluid/biomarkers, there is more tissue available for assays, skeletal maturity exists, a variety of joints can be used for modeling, and joints are of sufficient size for injectable therapies. Unfortunately, compared with their smaller animal models, they are more expensive, more difficult to house, have more genetic variation, are not typically genetically modifiable, assays/sequencing are challenging, and a veterinarian partner is typically required for studies.

**Figure 3.**
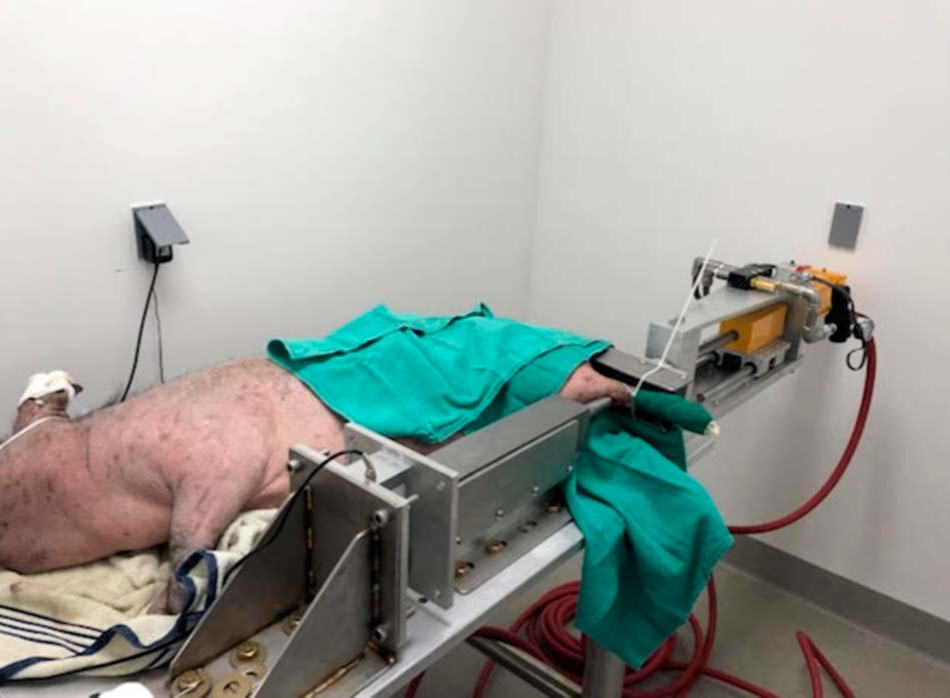
Porcine model used to create pilon fracture and demonstrate importance of articular reduction in porcine pilon model; decreased damage and PTOA when articular fragments are reconstructed. PTOA, posttraumatic arthritis.

Goetz et al used the benefits of a large animal to create a realistic large animal model with porcine pilon fractures that progressed to posttraumatic osteoarthritis. Their model demonstrated reproducible fracture production in a skeletally mature minipig ankle joint with similar morphology and cartilage thickness to humans. Fixation was performed after fractures, and the group with articular step off had increased histologic degeneration compared with the group with anatomic reduction. Overall, this model replicated the human condition well, but the PTOA in this model is slow to develop.^30^ Dekeyser et al also used a similar porcine animal model to demonstrate that articular fragment restoration is critical to mitigate posttraumatic osteoarthritis after pilon fractures created by compressed air impaction (Fig. 3).^31^

To get started with animal models for PTOA, it is important to divide steps into 3 phases. First is the feasibility phase, where you must identify a study question and your specific aims, contact an expert on the animal model of interest, engage a local colleague with animal model experience, and finally determine whether you can do this study at your institution. Once you have completed this phase, you can move onto the pilot phase where you develop your team (ie, veterinarians, PhD technicians), perform a couple of live animal experiments, troubleshoot, and then repeat. If resultant data are reproducible, you can move onto the final study phase. At this point, it is important to be aware of and apply for funding using your pilot data in advance of grant deadlines, be aware of the importance of documenting each step, routinely meet with your team, and complete the project within the planned timeline.

## 6. Conclusion

Orthopaedic trauma remains a leading cause of morbidity. However, the variability of the field presents challenges in research, especially evaluating pathophysiology mechanisms and testing therapies in a controlled setting. Preclinical animal models provide a partial solution by allowing for the study of numerous pathologies and treatments. As a result, animal models have been instrumental in understanding and treatments for posttraumatic arthritis, fracture healing in the setting of polytrauma, and fracture-related infection. These studies then lead to translational therapies, and research aimed at improvement of the human patient. As always, the continued humane use of preclinical animal models in the field of orthopaedic trauma remains at the forefront of the research design. Guidelines such as ARRIVE help to give a framework for ethically considerate research in partnership with animal support staff. The use of animal preclinical models is a privilege that can help us further improve the human condition.
